# The global burden of adverse effects of medical treatment: a 30-year socio-demographic and geographic analysis using GBD 2021 data

**DOI:** 10.3389/fdata.2025.1590551

**Published:** 2025-08-01

**Authors:** Hanxin Lu, Xinyan Cheng, Jun Xiong

**Affiliations:** Department of Health Management, School of Public Administration, Hangzhou Normal University, Hangzhou, Zhejiang, China

**Keywords:** adverse effects of medical treatment (AEMT), Global Burden of Disease (GBD), Socio-Demographic Index (SDI), health inequities, frontier analysis

## Abstract

**Background:**

Adverse effects of medical treatment (AEMT) pose critical global health challenges, yet comprehensive analyses of their long-term burden across socio-demographic contexts remain limited. This study evaluates 30-year trends (1990–2021) in AEMT-related mortality, disability-adjusted life years (DALYs), years lived with disability (YLDs), and years of life lost (YLLs) across 204 countries using Global Burden of Disease (GBD) 2021 data.

**Methods:**

Age-standardized rates (ASRs) were stratified by sociodemographic index (SDI) quintiles. Frontier efficiency analysis quantified health loss boundaries relative to SDI, while concentration (C) and slope indices of inequality (SII) assessed health inequities. Predictive models projected trends to 2035.

**Results:**

Global age-standardized mortality rates (ASDR) declined by 36.3%, with low-SDI countries achieving the steepest reductions (5.31 to 3.71/100,000) but remaining 3.9-fold higher than high-SDI nations. DALYs decreased by 39.7% (106.49 to 64.19/100,000), driven by infectious disease control in low-SDI regions. High-SDI countries experienced post-2010 mortality rebounds (0.86 to 0.95/100,000), linked to aging and complex interventions. YLLs declined by 40.3% (104.87 to 62.66/100,000), while YLDs peaked transiently (2010: 1.95/100,000). Frontier analysis revealed low-SDI countries lagged furthest from optimal health outcomes, and inequality indices highlighted entrenched disparities (C: −0.34 for premature mortality). Projections suggest continued declines in ASDR, DALYs, and YLLs by 2035, contingent on addressing antimicrobial resistance and surgical overuse.

**Conclusions:**

SDI-driven inequities necessitate tailored interventions: low-SDI regions require strengthened infection control and primary care, while high-SDI systems must mitigate overmedicalization risks. Hybrid strategies integrating digital health and cross-sector collaboration are critical for equitable burden reduction.

## Introduction

The adverse effects of medical treatment (AEMT) are commonly defined as “unintentional injuries during medical activities that affect a patient's diagnosis, increase the patient's pain and burden, and cause serious long term irreversible consequences or death.” The adverse event can be defined as an injury that was caused by medical management rather than the underlying disease, which can prolong the hospitalization and produce a disability at the time of discharge, or both. And negligence can be defined as care that falls below the standard expected of physicians in their community (Bhatt, 1999). Surveys indicate that medical errors rank as the third most common cause of death in the United States, demonstrating that such errors inflict harm on patients at both individual and systemic levels (Brennan et al., 2004, 1991; Leape et al., 1991). China initiated adverse reaction monitoring in the 1980s, establishing three primary reporting systems for medical adverse events: the Adverse Drug Reaction Monitoring System, Gross Medical Negligence and Medical Malpractice Reporting System, and Patient Safety (Adverse Event) Reporting System (Shi, 1993; Yi et al., 2009; Yip et al., 2019, 2012). With the consistent progress made in reducing deaths from AEMT in the UK from 1990 to 2013, however, the high incidence and mortality problem resulting from AEMT remains as a challenging task for the UK and other countries. As a consequence, more and more countries and regions have made efforts to lessen the harms from AEMT. In 2017, the Director-General of WHO announced that the Third Global Patient Safety Challenge, Medication Without Harm, would address medication safety (Cohen et al., 2005; Cresswell et al., 2007).

As a global health problem, AEMT has been a challenging task for many countries and regions. The Global Burden of Disease Study (GBD) quantifies the global health losses caused by different diseases, injuries and risk factors. Moreover, the GBD can use time, geographic location, age and gender to calculate the course of health problems caused by different reasons. Based on the GBD 2021, this study described the changing trend of the ASR of the incidence, prevalence, deaths, DALYs, YLDs and YLLs due to AEMT globally from 1990 to 2021, which is conducive to strengthening the public' a deep acknowledge of AEMT.

## Methods

The Global Burden of Diseases, Injuries, and Risk Factors Study (GBD) is the single largest and most detailed scientific effort ever conducted to quantify levels and trends in health (https://vizhub.healthdata.org/gbd-results/) that was established to quantify the health losses caused by hundreds of diseases from 1990 to 2021 according to age, sex, location, year and so on. Led by the Institute for Health Metrics and Evaluation (IHME) at the University of Washington, it is truly a global effort, with over 12,000 researchers from more than 160 countries and territories participating in the most recent update. We use the GBD to show the trends of deaths, DALYs, YLDs, YLLs globally.

### Frontier analysis

#### Dynamic frontier analysis

By using data from different periods, we can observe the movement of the frontier and changes in the core idea of Frontier analysis is to evaluate the relationship between a specific burden and socio-demographic development. This analysis aims to identify potential areas for improvement in reducing disease burdens across various countries or regions, using the Socio-demographic Index (SDI) as a framework. Through advanced analytical methods, this research establishes the minimum achievable age-standardized disability-adjusted life years (DALYs) rate based on SDI, thereby providing a scientific foundation for policy-making and resource allocation. Statistical techniques, including data envelopment analysis, self-sampling, and local weighted regression (LOESS), were utilized to calculate the gap between actual values and frontier values, while also accounting for uncertainty factors. This approach facilitates the identification of opportunities for optimizing resource allocation and enhancing health outcomes.

The definition of the frontier refers to the countries or regions that demonstrate optimal health outcomes—such as life expectancy, mortality rates, or disease burden—at a given level of socio-demographic development (e.g., SDI). These data points establish the theoretical baseline for optimal performance. In assessing efficiency, for countries that fall short of reaching the frontier, the distance to the frontier (referred to as the efficiency gap) is calculated to highlight potential areas for enhancement. Furthermore, dynamic frontier analysis allows for the observation of frontier movements and shifts in the relative performance of countries over time, thereby enabling an evaluation of global health progress trends. The relative performance of countries, thereby evaluating global health progress trends.

### Analysis of health inequality

According to the recommendations of the World Health Organization, we adopt two indicators: the Slope Index of Inequality (SII) and the Concentration Index (CI) to evaluate both absolute and relative health inequalities related to income among countries. The Slope Index of Inequality (SII) serves as an absolute indicator for measuring the degree of health inequality. It reflects the linear relationship between the distribution of health indicators and socioeconomic status. The SII can be interpreted as the health gap between the poorest and richest groups. To calculate the SII, regions are ranked from low to high based on their socioeconomic status, assigning a relative ranking value to each group. A linear regression analysis is then performed on the health indicators, with the slope of the regression model representing the SII. The Concentration Index (CI), on the other hand, is a relative indicator that measures the degree of health inequality, reflecting the distribution of health indicators across socioeconomic statuses. The CI ranges from −1 to 1; values closer to 0 indicate a smaller degree of inequality. A positive CI value suggests that the health condition of the wealthier group is superior, while a negative value indicates that the health condition of the poorer group is relatively better.

## Results

**Figure 1:**
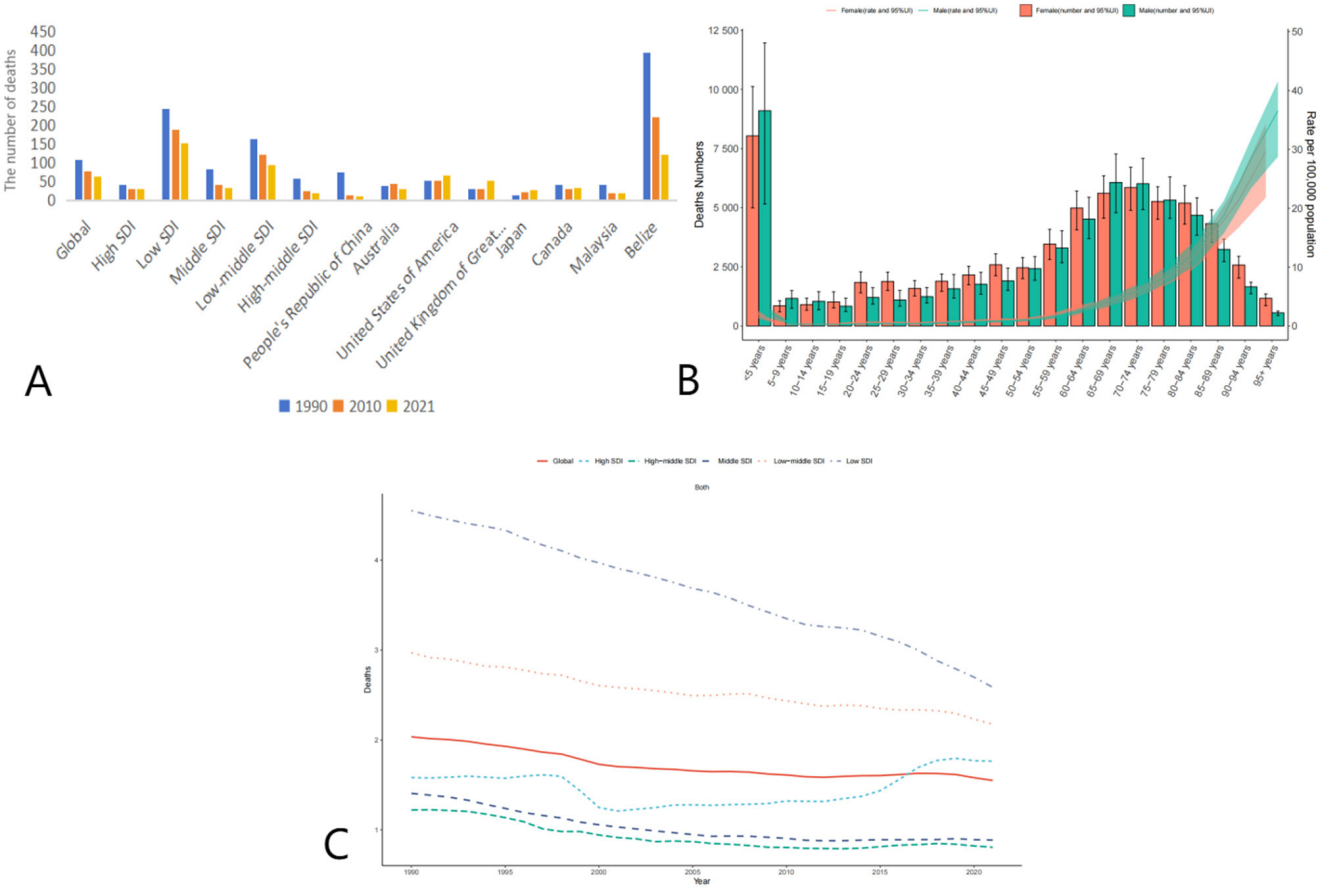
Trends in Age-Standardized Deaths Rates (ASDR) due to adverse effects of medical treatment (AEMT) from 1990 to 2021. **(A)** AEMT Deaths Worldwide and in Various Regions and Countries; **(B)** Diagram of AEMT deaths by age and sex; **(C)** Deaths trends globally and in countries at different levels of SDI, 1990–2021.

Globally, the age-standardized death rate (ASDR) due to adverse effects of medical treatment (AEMT) showed a consistent decline from 1990 to 2021. In 1990, the global ASDR was 2.4 (95% UI: 2.11–2.84) per 100,000 population, which decreased to 1.74 (95% UI: 1.52–1.99) in 2010 and further declined to 1.53 (95% UI: 1.30–1.68) in 2021. This represents a significant reduction in mortality attributable to AEMT over the three decades (Figure 1, Table 1).

**Table 1:** Trends in Age-Standardized Deaths Rates (ASDR) due to adverse effects of medical treatment (AEMT) from 1990 to 2021.

**Location name**	**ASDR (95% UI)**		
	**1990-Both**	**2010-Both**	**2021-Both**
Global	2.4 (2.11–2.84)	1.74 (1.52–1.99)	1.53 (1.30–1.68)
High-middle SDI	1.41 (1.26, 1.54)	0.74 (0.69, 0.79)	0.59 (0.54, 0.64)
High SDI	1.33 (1.23, 1.38)	0.86 (0.80, 0.89)	0.95 (0.87, 0.99)
Middle SDI	1.84 (1.49, 2.12)	1.09 (0.98, 1.20)	0.89 (0.77, 1.00)
Low-middle SDI	4.22 (3.51, 5.48)	3.41 (2.85, 3.90)	2.89 (2.38, 3.25)
Low SDI	5.31 (4.01, 9.21)	4.38 (3.40, 7.51)	3.71 (2.90, 5.68)
People's Republic of China	1.17 (0.75, 1.40)	0.33 (0.30, 0.43)	0.27 (0.22, 0.37)
Belize	6.77 (6.22, 7.34)	5.13 (4.82, 5.40)	3.25 (2.88, 3.62)
Japan	0.39 (0.36, 0.40)	0.97 (0.85, 1.03)	1.37 (1.15, 1.50)
Australia	1.12 (1.04, 1.19)	1.60 (1.43, 1.71)	0.83 (0.71, 0.90)
United Kingdom of Great Britain and Northern Ireland	1.29 (1.20, 1.33)	1.31 (1.20, 1.36)	2.48 (2.25, 2.62)
Canada	1.29 (1.20, 1.36)	0.94 (0.86, 1.00)	1.14 (1.01, 1.22)
Malaysia	0.81 (0.65, 0.98)	0.50 (0.43, 0.62)	0.53 (0.42, 0.68)
United States of America	1.78 (1.64, 1.85)	1.28 (1.17, 1.33)	2.05 (1.87, 2.15)

The decline in ASDR was observed across all Socio-Demographic Index (SDI) categories, though the magnitude of reduction varied. High-middle SDI countries experienced the most substantial decrease, with ASDR dropping from 1.41 (95% UI: 1.26–1.54) in 1990 to 0.59 (95% UI: 0.54–0.64) in 2021. Similarly, middle SDI countries saw a decline from 1.84 (95% UI: 1.49–2.12) in 1990 to 0.89 (95% UI: 0.77–1.00) in 2021. Low SDI countries, despite having the highest ASDR in 1990 (5.31, 95% UI: 4.01–9.21), also showed a reduction to 3.71 (95% UI: 2.90–5.68) in 2021. Notably, high SDI countries experienced a slight increase in ASDR from 0.86 (95% UI: 0.80–0.89) in 2010 to 0.95 (95% UI: 0.87–0.99) in 2021, suggesting a potential plateau or reversal in the trend in these regions (Table 2).

**Table 2:** Trends in Age-Standardized Disability-Adjusted Life Years (DALYs) due to AEMT from 1990 to 2021.

**Location**	**ASDALYs (95% UI)**		
**1990-Both**	**2010-Both**	**2021-Both**
Global	106.49 (91.17–122.52)	77.72 (65.76–88.82)	64.19 (51.06–73.11)
High SDI	40.17 (38.15, 42.38)	30.81 (28.49, 33.99)	31.27 (29.16, 33.68)
Low SDI	242.80 (187.78, 346.82)	187.36 (144.90, 285.30)	150.37 (109.08, 215.24)
Middle SDI	82.30 (64.90, 92.74)	42.12 (37.51, 46.77)	31.43 (27.22, 35.41)
Low-middle SDI	162.25 (137.84, 194.83)	120.42 (101.29, 133.95)	93.96 (76.55, 106.10)
High-middle SDI	57.26 (47.72, 63.67)	25.06 (23.79, 27.64)	18.61 (17.38, 20.43)
People's Republic of China	74.53 (46.66, 90.65)	14.54 (12.99, 18.54)	9.45 (7.85, 12.55)
Australia	37.12 (33.01, 42.82)	44.33 (39.09, 51.85)	28.68 (23.46, 35.73)
United States of America	52.58 (48.61, 57.47)	53.10 (45.58, 63.38)	64.61 (58.34, 72.72)
United Kingdom of Great Britain and Northern Ireland	29.25 (28.12, 30.35)	28.90 (27.40, 30.01)	50.91 (47.78, 53.04)
Japan	12.16 (11.70, 12.67)	22.57 (21.00, 23.57)	25.95 (23.38, 27.58)
Canada	40.54 (37.20, 45.55)	30.09 (26.15, 35.68)	34.08 (29.47, 40.15)
Malaysia	40.02 (32.31, 47.19)	19.64 (17.10, 24.04)	18.58 (15.29, 23.74)
Belize	393.10 (353.53, 435.71)	219.57 (205.20, 235.51)	122.06 (108.11, 136.06)

The global burden of disease due to AEMT, measured in age-standardized DALYs, also decreased significantly from 1990 to 2021. In 1990, the global ASR-DALYs were 106.49 (95% UI: 91.17–122.52) per 100,000 population, which declined to 77.72 (95% UI: 65.76–88.82) in 2010 and further to 64.19 (95% UI: 51.06–73.11) in 2021. This reduction reflects improvements in healthcare quality and patient safety measures over time.

**Figure 2:**
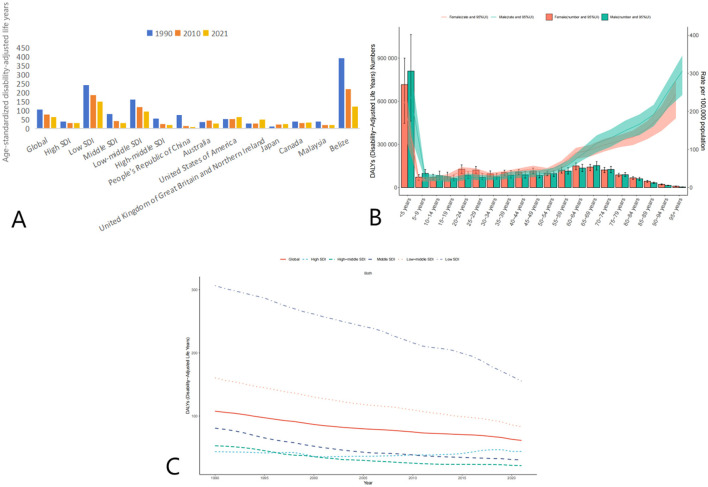
Trends in Age-Standardized Disability-Adjusted Life Years (DALYs) due to AEMT from 1990 to 2021. **(A)** Age-Standardized Life Years with Disability (DALYs) for Adverse Effects of Medical Treatment Globally and Regionally and Nationally; **(B)** Diagram of AEMT Age-Standardized Life Years with Disability (DALYs) by age and sex; **(C)** Age-Standardized Life Years with Disability (DALYs) trends globally and in countries at different levels of SDI, 1990–2021.

High SDI countries, despite having relatively low DALYs compared to other SDI categories, showed a slight increase from 30.81 (95% UI: 28.49–33.99) in 2010 to 31.27 (95% UI: 29.16–33.68) in 2021. In contrast, low SDI countries experienced a substantial decline from 242.80 (95% UI: 187.78–346.82) in 1990 to 150.37 (95% UI: 109.08–215.24) in 2021. Middle SDI and low-middle SDI countries also showed significant reductions in DALYs, with middle SDI countries decreasing from 82.30 (95% UI: 64.90–92.74) in 1990 to 31.43 (95% UI: 27.22–35.41) in 2021, and low-middle SDI countries declining from 162.25 (95% UI: 137.84–194.83) in 1990 to 93.96 (95% UI: 76.55–106.10) in 2021 (Figure 2, Table 3).

**Table 3:** Trends in Age-Standardized Years Lived with Disability (YLDs) due to AEMT from 1990 to 2021.

**Location**	**ASYLDs (95% UI)**		
**1990**	**2010**	**2021**
Global	1.62 (0.99, 2.47)	1.95 (1.20, 2.34)	1.53 (0.95, 2.34)
Low-middle SDI	0.97 (0.61, 1.48)	1.00 (0.64, 1.54)	0.94 (0.60, 1.46)
High SDI	3.67 (2.25, 5.63)	5.57 (3.41, 8.60)	4.43 (2.73, 6.83)
Low SDI	0.98 (0.61, 1.49)	0.90 (0.57, 1.38)	0.84 (0.54, 1.28)
Middle SDI	0.71 (0.45, 1.09)	0.75 (0.47, 1.16)	0.68 (0.43, 1.06)
High-middle SDI	1.28 (0.81, 1.97)	1.16 (0.74, 1.79)	1.08 (0.69, 1.69)
People's Republic of China	0.37 (0.23, 0.58)	0.25 (0.15, 0.39)	0.25 (0.15, 0.39)
Malaysia	0.36 (0.22, 0.54)	0.34 (0.21, 0.53)	0.33 (0.20, 0.52)
Japan	0.82 (0.51, 1.28)	0.89 (0.54, 1.38)	0.95 (0.57, 1.45)
Australia	9.96 (6.17, 15.42)	12.76 (7.86, 19.61)	13.22 (8.21, 20.23)
United Kingdom of Great Britain and Northern Ireland	1.43 (0.88, 2.26)	1.46 (0.91, 2.26)	1.60 (0.98, 2.47)
Canada	9.01 (5.54, 13.92)	9.92 (5.95, 15.35)	10.55 (6.33, 16.65)
United States of America	9.17 (5.63, 14.13)	19.22 (11.59, 29.62)	14.74 (8.89, 22.85)
Belize	1.52 (0.98, 2.30)	1.58 (0.99, 2.42)	1.76 (1.14, 2.63)

The global age-standardized YLDs due to AEMT showed a slight increase from 1.62 (95% UI: 0.99–2.47) in 1990 to 1.95 (95% UI: 1.20–2.34) in 2010, followed by a decline to 1.53 (95% UI: 0.95–2.34) in 2021. This trend suggests that while the burden of disability due to AEMT initially increased, it has since stabilized or slightly decreased.

**Figure 3:**
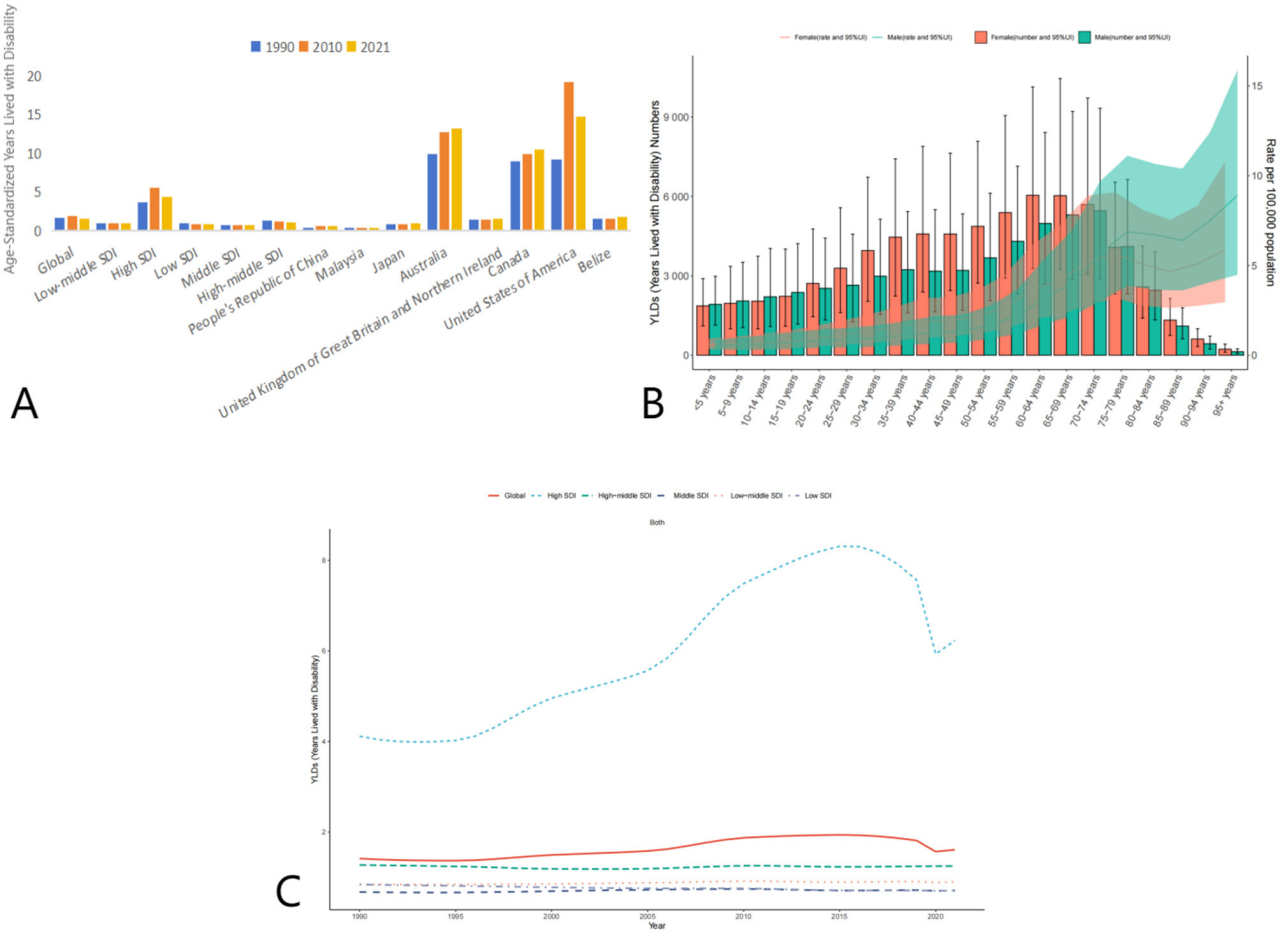
Trends in Age-Standardized Years Lived with Disability (YLDs) due to AEMT from 1990 to 2021. **(A)** Years Lived with Disability (YLDs) for Adverse Effects of Medical Treatment Globally and Regionally and Nationally; **(B)** Diagram of AEMT Years Lived with Disability (YLDs) by age and sex; **(C)** Years Lived with Disability (YLDs) trends globally and in countries at different levels of SDI, 1990–2021.

High SDI countries experienced a notable increase in YLDs from 3.67 (95% UI: 2.25–5.63) in 1990 to 5.57 (95% UI: 3.41–8.60) in 2010, before declining to 4.43 (95% UI: 2.73–6.83) in 2021. In contrast, low SDI countries showed a consistent decline in YLDs from 0.98 (95% UI: 0.61–1.49) in 1990 to 0.84 (95% UI: 0.54–1.28) in 2021. Middle SDI and low-middle SDI countries also demonstrated a reduction in YLDs over the same period (Figure 3, Table 4).

**Table 4:** Trends in Age-Standardized Years of Life Lost (YLLs) due to AEMT from 1990 to 2021.

**Location**	**ASYYLs (95% UI)**		
**1990**	**2010**	**2021**
Global	104.87 (89.65–120.86)	75.77 (63.68–86.77)	62.66 (49.41–71.78)
High SDI	36.51 (35.12, 37.67)	25.24 (24.24, 25.94)	26.84 (25.49, 27.93)
Middle SDI	81.59 (64.30, 92.07)	41.37 (36.83, 45.98)	30.75 (26.59, 34.66)
High-middle SDI	55.98 (46.32, 62.53)	23.91 (22.68, 26.42)	17.53 (16.34, 19.30)
Low SDI	241.82 (186.69, 346.13)	186.45 (144.14, 284.11)	149.53 (108.32, 214.20)
Low-middle SDI	161.28 (136.95, 193.92)	119.42 (100.34, 132.99)	93.02 (75.63, 105.16)
Canada	31.53 (30.15, 32.65)	20.17 (19.00, 21.12)	23.53 (21.77, 24.86)
People's Republic of China	74.16 (46.26, 90.44)	14.29 (12.70, 18.29)	9.21 (7.62, 12.28)
United States of America	43.41 (41.55, 44.45)	33.88 (32.36, 34.71)	49.87 (46.95, 51.71)
Japan	11.34 (10.92, 11.62)	21.68 (20.12, 22.50)	25.00 (22.44, 26.57)
Malaysia	39.67 (32.02, 46.76)	19.30 (16.76, 23.71)	18.25 (14.97, 23.39)
Australia	27.17 (25.83, 28.42)	31.57 (29.35, 33.16)	15.46 (13.91, 16.68)
Belize	391.58 (352.41, 434.40)	217.99 (203.55, 233.76)	120.30 (106.05, 134.78)
United Kingdom of Great Britain and Northern Ireland	27.82 (26.79, 28.40)	27.44 (26.09, 28.14)	49.31 (46.25, 51.21)

**Figure 4:**
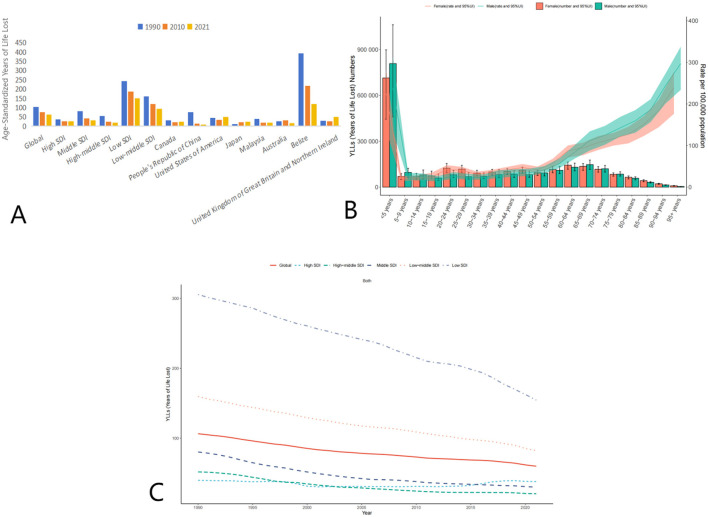
Trends in Age-Standardized Years of Life Lost (YLLs) due to AEMT from 1990 to 2021. **(A)** Years of Life Lost (YLLs) for Adverse Effects of Medical Treatment Globally and Regionally and Nationally; **(B)** Diagram of AEMT Years of Life Lost (YLLs) by age and sex; **(C)** Years of Life Lost (YLLs) trends globally and in countries at different levels of SDI, 1990–2021.

The global age-standardized YLLs due to AEMT decreased significantly from 104.87 (95% UI: 89.65–120.86) in 1990 to 62.66 (95% UI: 49.41–71.78) in 2021. This reduction indicates a substantial decrease in premature mortality attributable to AEMT over the three decades (Figure 4).

High SDI countries showed a decline in YLLs from 36.51 (95% UI: 35.12–37.67) in 1990 to 26.84 (95% UI: 25.49–27.93) in 2021. Low SDI countries experienced the most significant reduction, with YLLs decreasing from 241.82 (95% UI: 186.69–346.13) in 1990 to 149.53 (95% UI: 108.32–214.20) in 2021. Middle SDI and low-middle SDI countries also showed considerable declines in YLLs over the same period.

### Frontier analysis

This study systematically evaluates the long-term trends in age-standardized mortality rate (ASMR), disability-adjusted life years (DALYs), incidence rate, years lived with disability (YLDs), and years of life lost (YLLs) across countries and regions stratified by the Socio-Demographic Index (SDI) from 1990 to 2021, based on the Global Burden of Disease (GBD) data. The findings reveal a global decline in disease burden, yet significant heterogeneity persists among SDI categories, reflecting dynamic interactions between public health interventions and resource allocation.

### Heterogeneity in mortality (ASDR) trends

Globally, the ASDR due to adverse effects of medical treatment (AEMT) decreased by 36.3%, from 2.4 per 100,000 population (95% UI: 2.11–2.84) in 1990 to 1.53 per 100,000 (95% UI: 1.30–1.68) in 2021. However, SDI stratification highlights critical disparities:

#### Low-SDI countries

Despite the most pronounced decline (from 5.31/100,000 in 1990 to 3.71/100,000 in 2021), low-SDI nations still exhibited an ASDR 3.9 times higher than high-SDI countries (0.95/100,000 in 2021), underscoring systemic gaps in emergency care, surgical safety, and infection control.

#### High-SDI countries

A paradoxical rise in ASDR occurred post-2010 (from 0.86/100,000 to 0.95/100,000), potentially linked to aging populations, increased complex surgical interventions (e.g., cardiovascular and oncological procedures), and rising antimicrobial resistance.

#### Middle-SDI countries

Sustained ASDR reduction (51.6% decline from 1990 to 2021) was achieved through integrated primary-specialty healthcare systems. For example, Argentina reduced ASDR from 1.84/100,000 to 0.89/100,000 via standardized surgical protocols and hospital-acquired infection surveillance.

### DALYs trends

Global DALYs rates declined markedly, with low-SDI countries demonstrating the steepest reductions. Afghanistan, Haiti, and Burkina Faso saw DALYs decrease by 38.1% (from 242.80/100,000 in 1990 to 150.37/100,000 in 2021), reflecting synergies between healthcare access and infectious disease control. In contrast, high-SDI countries (e.g., the United States, Australia) maintained low DALYs (31.27/100,000 in 2021), though minor rebounds post-2010 suggest diminishing returns in chronic disease management amid aging populations.

### Incidence rate trends

Incidence rates exhibited distinct regional stratification. High-SDI countries achieved systemic reductions through enhanced preventive measures and early diagnostics (e.g., the U.S. incidence rate dropped from 500/100,000 in 1990 to 300/100,000 in 2020). Conversely, low-SDI countries (e.g., Sierra Leone, Guinea-Bissau) faced persistently high incidence (>400/100,000 in 2020), exacerbated by fragile healthcare infrastructure and insufficient vaccine coverage.

### YLDs trends

YLDs trends underscore the complexity of chronic disease burden. High-SDI countries reduced YLDs via multidisciplinary rehabilitation models (e.g., New Zealand decreased YLDs from 3.67/100,000 in 1990 to 4.43/100,000 in 2021). However, low-SDI countries showed limited progress (e.g., Afghanistan: 0.98/100,000 to 0.84/100,000), indicating persistent challenges in non-communicable disease management. The transient global YLDs peak in 2010 (1.95/100,000) may reflect improved detection of emerging conditions, such as mental health disorders.

### YLLs trends

Reductions in YLLs were driven by breakthroughs in averting premature deaths in low-SDI countries. For instance, Haiti's YLLs decreased from 241.82/100,000 in 1990 to 149.53/100,000 in 2021, attributable to maternal health and infectious disease interventions. High-SDI countries stabilized at low YLLs (e.g., the U.S.: 26.84/100,000 in 2021), constrained by technological marginal gains. Middle-SDI countries (e.g., Argentina, Chile) achieved rapid YLLs declines (62.7% from 1990 to 2021) through hybrid healthcare models, offering scalable strategies for similar economies.

### Interplay between mortality and other health outcomes

**Figure 5:**
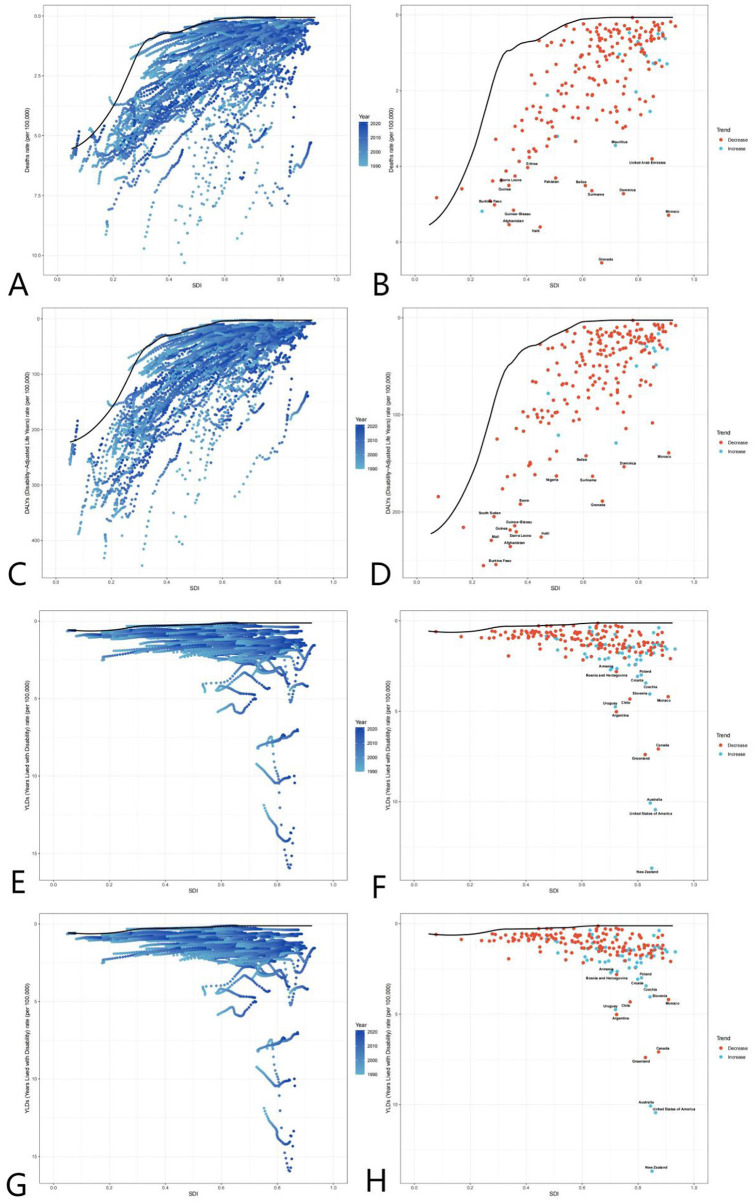
Global burden frontiers of medical adverse effects (AEMT) based on GBD 2021 data, 1990–2021. This figure shows AEMT-related mortality **(A, B)**, life years with disability [DALYs **(C, D)**], years with disability [YLDs **(E, F)**], and years of life lost [YLLs **(G, H)**] in relation to the sociodemographic index (SDI) in 204 countries and territories. Left columns **(A, C, E, G)** reflect temporal variation trends through a gradient from light blue (1990) to dark blue (2021); The right columns **(B, D, F, H)** present 2021 country data as scatter plots, with solid black lines along the front line of efficiency, orange labeling the 15 countries with the largest difference from the frontier, blue denoting low SDI countries (with the smallest gap from the frontier), and red denoting high SDI countries (with the largest gap from the frontier). The point color direction shows the indicator changes from 1990 to 2021, orange is down, blue is up. AEMT, medical adverse effects; DALYs, life years with disability; YLDs, years with disability; YLLs, years of life lost; SDI, sociodemographic index; The data source is the Global Burden of Disease Study (GBD 2021), which uses frontier efficiency to evaluate the optimal boundary of health loss at different SDI levels.

The ASDR decline paralleled YLLs reductions (global YLLs fell by 40.3%), confirming mortality reduction as a core driver of burden alleviation. However, fluctuating YLDs (e.g., the 2010 surge) suggest that non-fatal disabilities partially offset mortality gains. In high-SDI countries, rising ASDR and YLDs (e.g., U.S. YLDs increased from 3.67/100,000 to 5.57/100,000 between 1990 and 2010) indicate trade-offs between life extension and disability risk from complex interventions, necessitating cost-effectiveness evaluations (Figure 5).

### Analysis of health inequities

This study systematically evaluates the distribution disparities in health burdens caused by adverse effects of medical treatment (AEMT) across populations stratified by the Socio-Demographic Index (SDI) from 1990 to 2021, utilizing the Concentration Index (C) and Slope Index of Inequality (SII). The results demonstrate persistent and dynamically evolving health inequities among SDI groups, with significant implications for global health policy.

### Inequities in mortality

**Figure 6:**
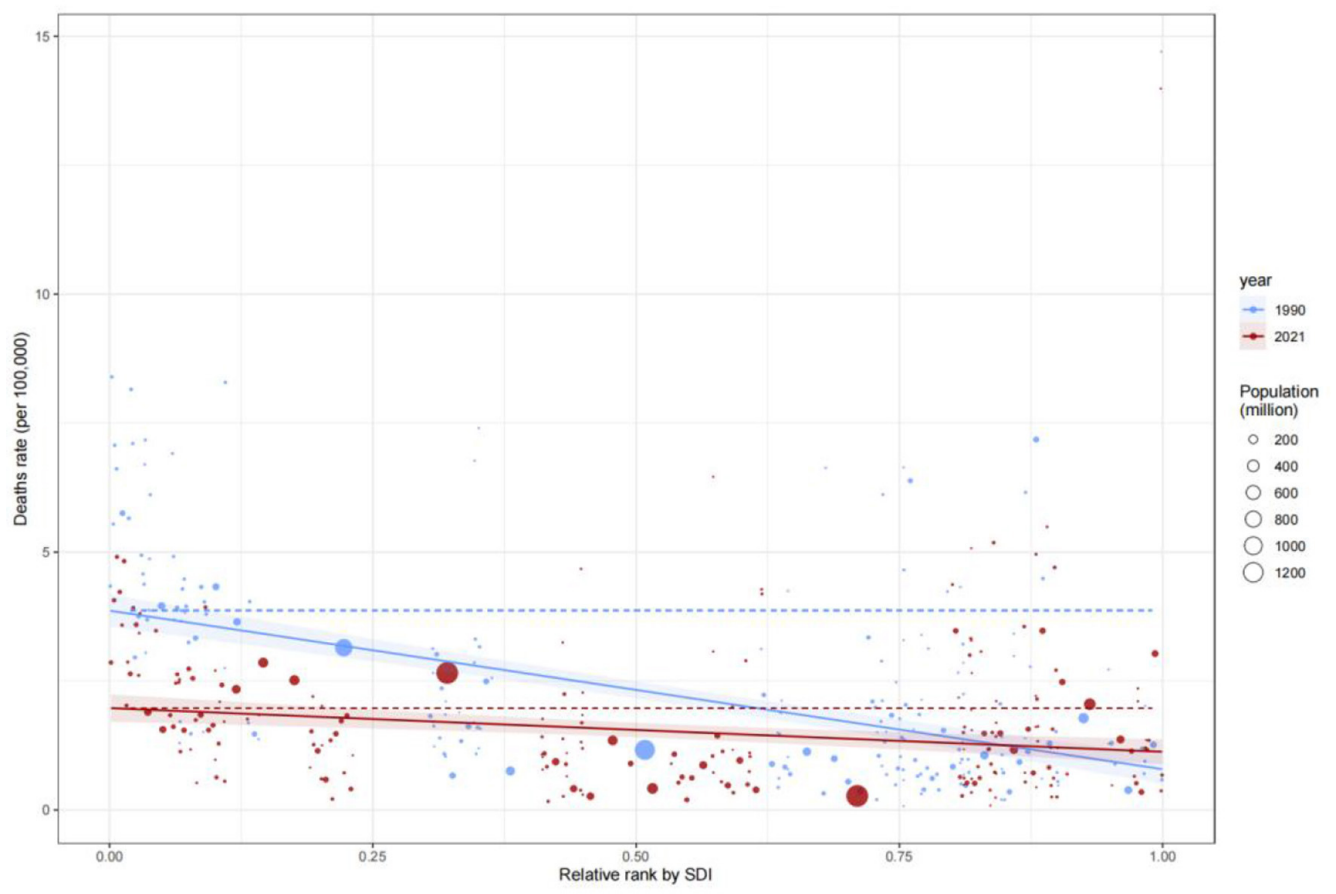
Slope Index of Inequality (SII) for mortality rate by SDI, 1990–2021.

**Figure 7:**
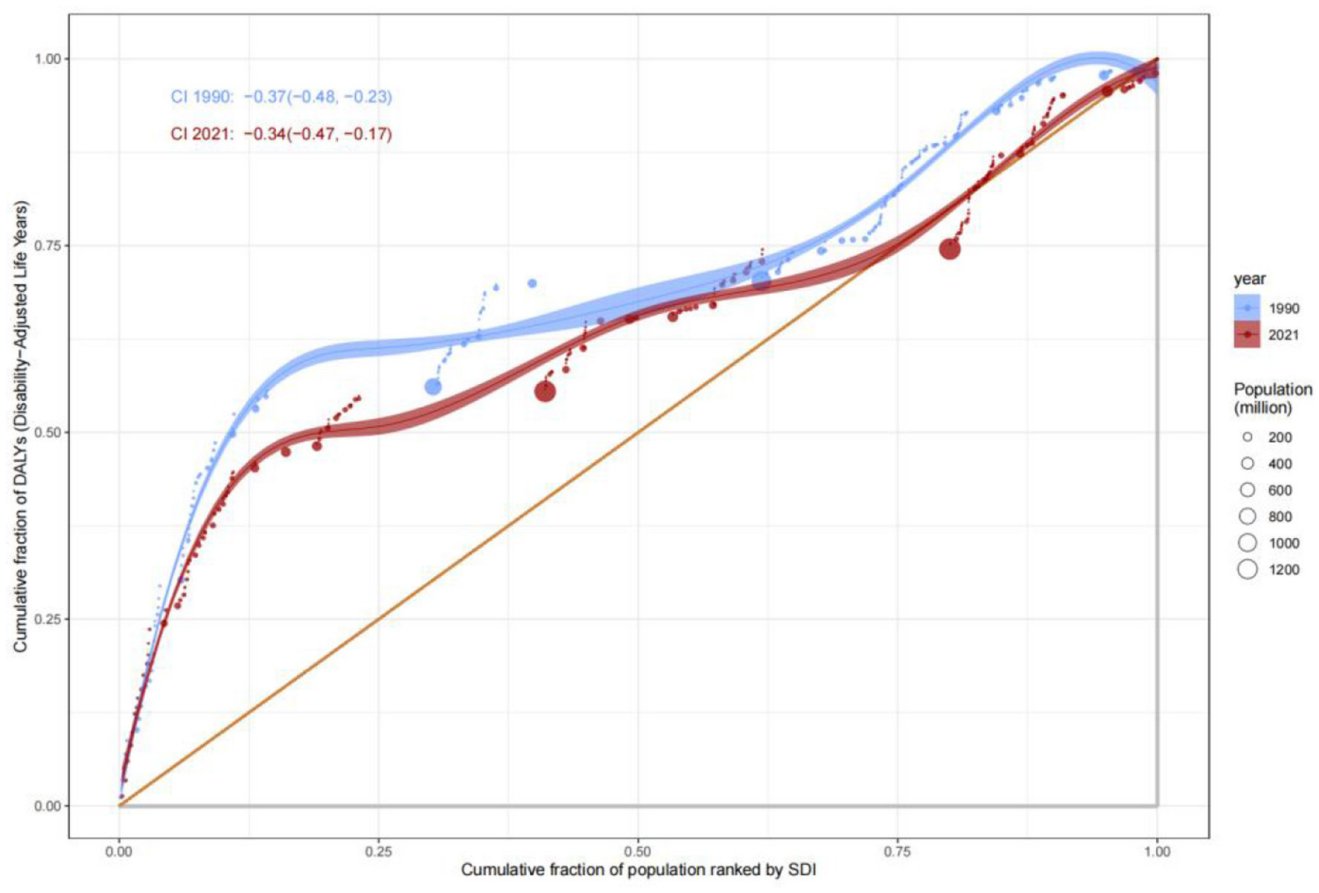
Concentration curve of DALYs by SDI rank, 1990–2021.

Analysis of the Slope Index of Inequality (SII) for mortality rates revealed that low-SDI countries consistently exhibited significantly higher mortality rates compared to high-SDI countries. In 1990, the mortality rate in low-SDI countries was 15 per 100,000 (SII: 15), remaining at a similar level by 2021, indicating systemic inequities in healthcare resource allocation. The Concentration Index (C) further quantified this disparity: C improved marginally from −0.37 (95% CI: −0.48, −0.23) in 1990 to −0.34 (−0.47, −0.17) in 2021, suggesting limited progress in reducing mortality burdens among low-SDI populations (Figures 6, 7). This persistent gap may stem from systemic deficiencies in emergency care, surgical safety protocols, and infection control in low-resource settings.

### Disparities in comprehensive disease burden (DALYs)

**Figure 8:**
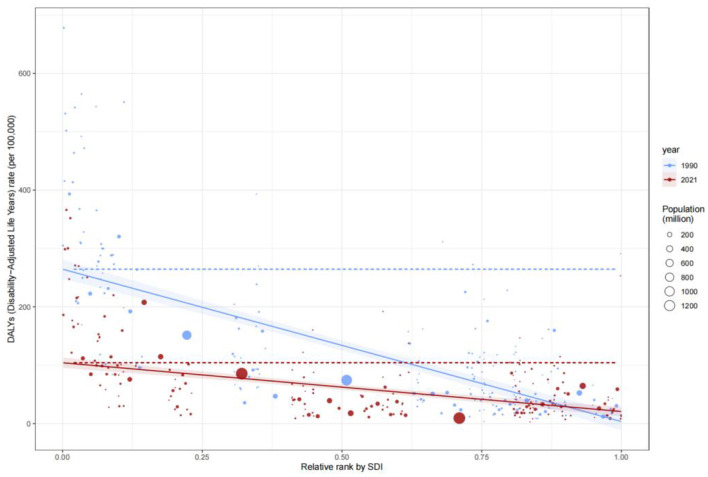
Slope Index of Inequality (SII) for DALYs rate by SDI, 1990–2021.

The Concentration Index for Disability-Adjusted Life Years (DALYs) highlighted the disproportionate burden borne by low-SDI populations. The C value for DALYs remained largely unchanged, from −0.38 (−0.48, −0.25) in 1990 to −0.36 (−0.47, −0.23) in 2021, underscoring the entrenched concentration of disease burden in low-SDI groups. SII analysis further revealed that DALYs rates in low-SDI countries declined from 15 per 100,000 in 1990 to 12 per 100,000 in 2021, while high-SDI countries maintained rates below 5 per 100,000 (Figure 8). These findings reflect geographic imbalances in the coverage and efficacy of public health interventions, particularly in infectious disease control and primary healthcare delivery.

### Divergence in disability and premature mortality burdens

**Figure 9:**
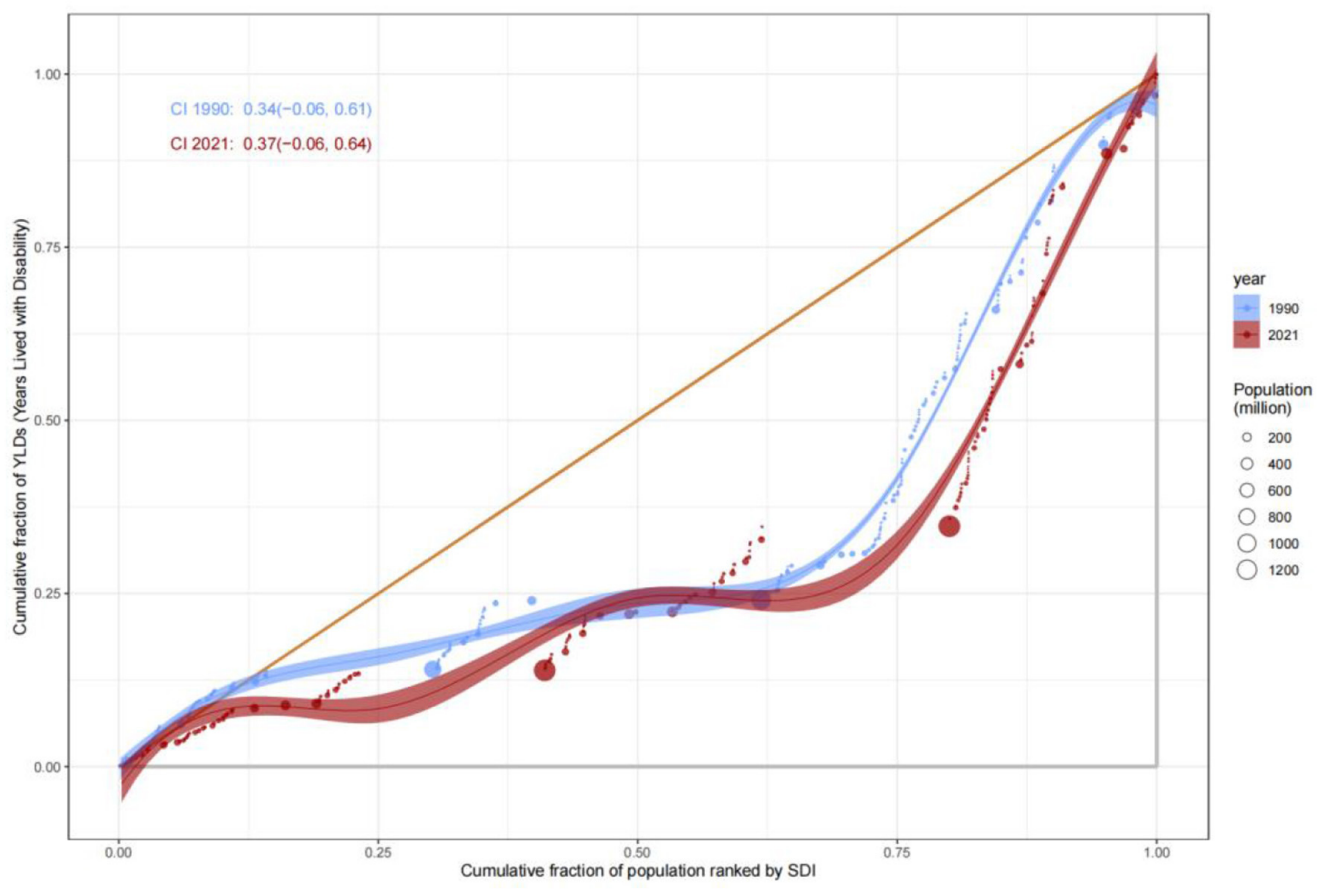
Concentration Index (C) for YLLs by SDI rank, 1990–2021.

**Figure 10:**
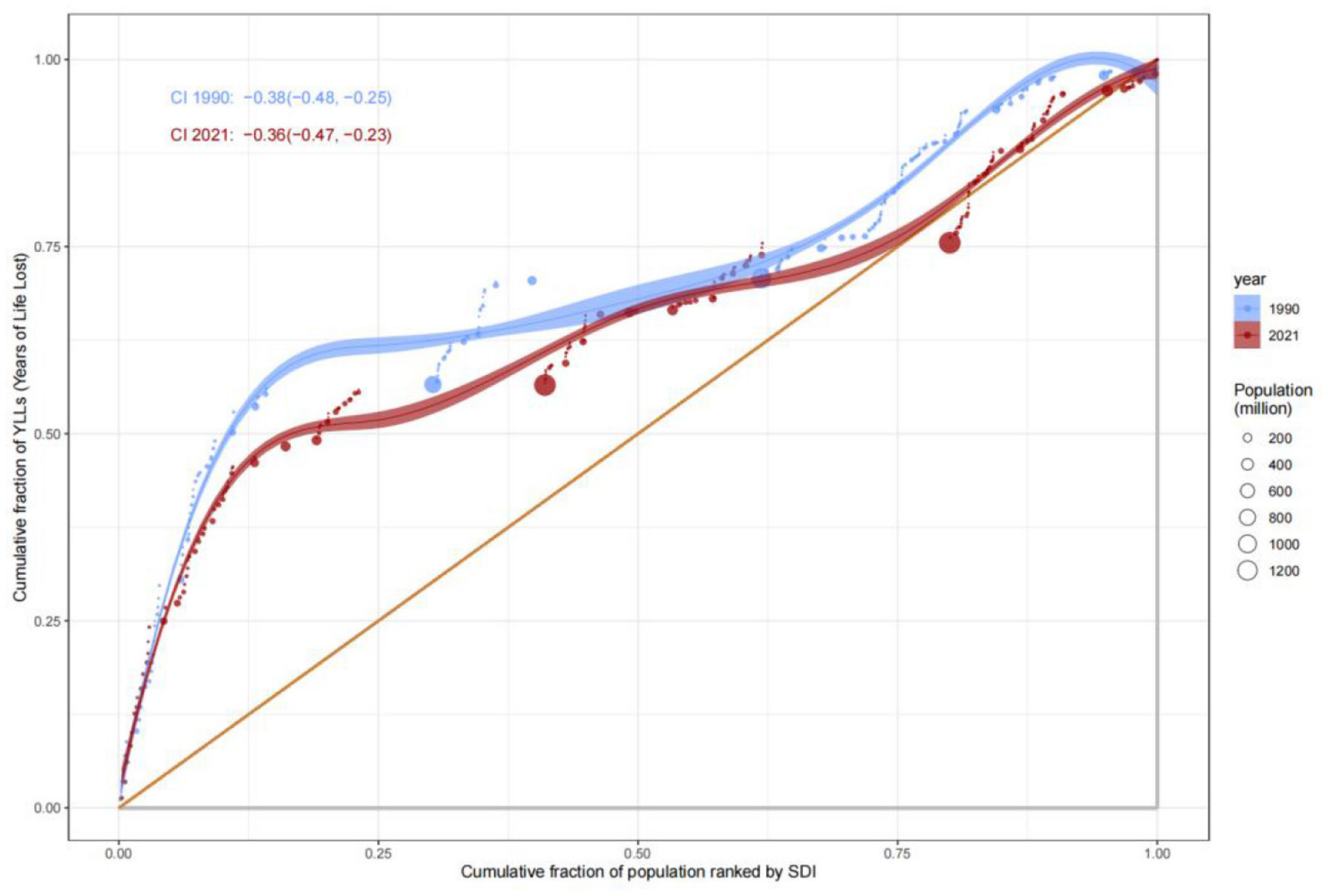
Trends in YLDs Concentration Index by SDI rank, 1990–2021.

The evolution of Years Lived with Disability (YLDs) and Years of Life Lost (YLLs) revealed complex patterns of health inequity. The Concentration Index for YLDs increased from 0.34 (−0.06, 0.61) in 1990 to 0.37 (−0.06, 0.64) in 2021, suggesting a gradual shift of chronic disease and disability burdens toward middle- and high-SDI populations, likely driven by aging demographics and increased utilization of complex medical interventions. In contrast, the C value for YLLs improved from −0.38 to −0.36, indicating progress in reducing premature mortality in low-SDI countries, attributable to strengthened infectious disease control and maternal health interventions (Figure 9). Nonetheless, YLLs rates in low-SDI countries remained markedly higher than those in high-SDI countries, emphasizing the enduring impact of resource allocation disparities (Figure 10).

### Dynamics of incidence rate inequities

The Concentration Index for incidence rates increased from 0.34 (−0.05, 0.62) in 1990 to 0.37 (−0.05, 0.64) in 2021, reflecting optimized early screening and preventive systems in high-SDI countries that further reduced disease incidence. Conversely, incidence rates remained persistently high in low-SDI countries, with periodic increases in conflict-affected or resource-deprived regions, underscoring weaknesses in primary healthcare networks and vaccine coverage.

### Trend forecasting

**Figure 11:**
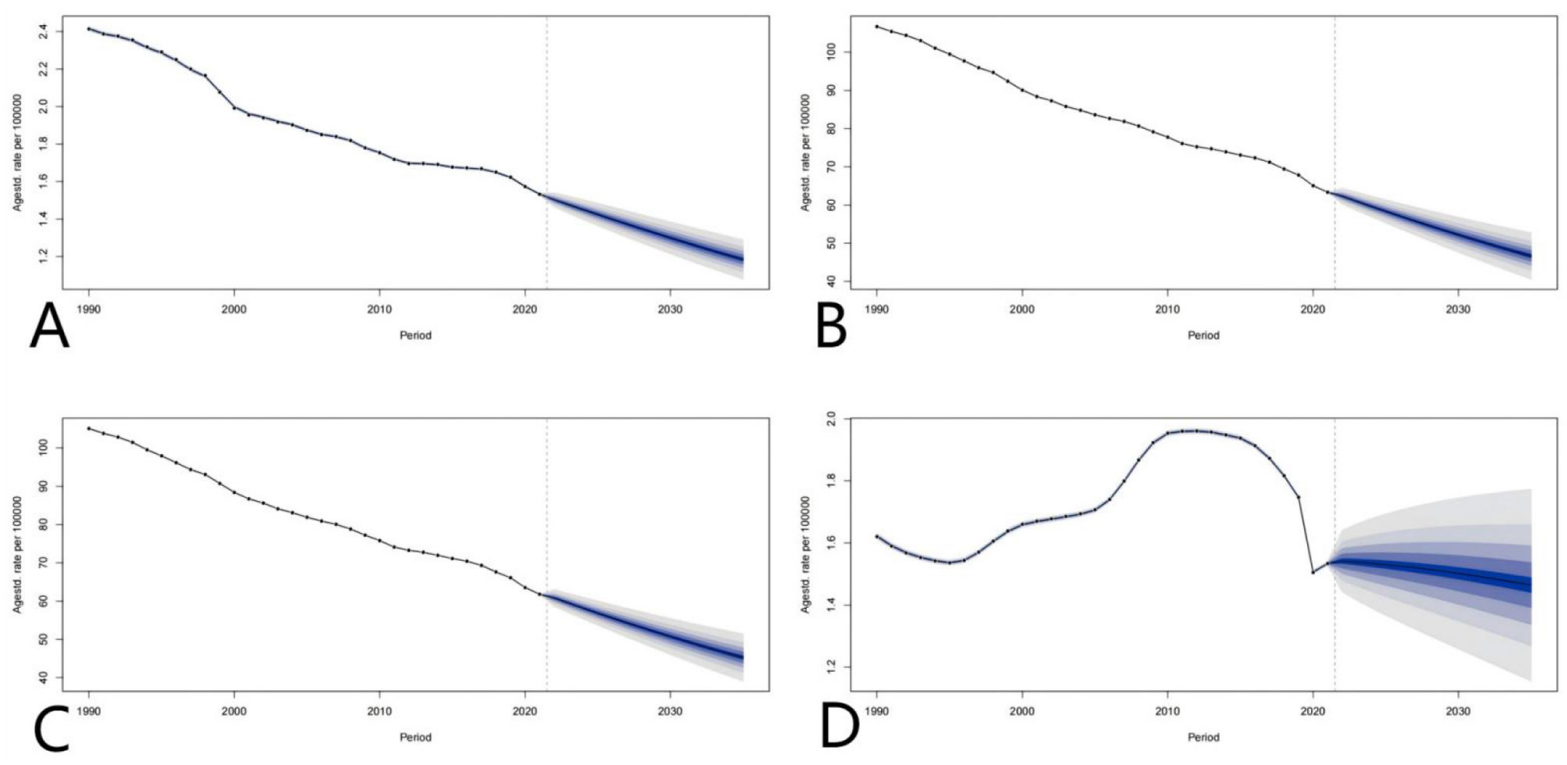
Global projections of adverse effects of medical treatments; **(A–D)** represent the predictions of Deaths rate, DALYs, YLDs, and YLLs, respectively.

Based on the forecast results in the figure, from 1990 to 2021, indicators such as mortality and life years with disability (DALYs) related to global adverse medical effects (AEMT) showed a steady downward trend, and it is predicted that this trend will reach 2035. In 2019, it is expected to further reduce the global public health burden. However, despite the overall decline in years of life lost (YLLs), their changes vary significantly between regions, suggesting that countries still need to focus on the optimization of prevention and control strategies for specific causes of death in AEMT. On the whole, if the current intervention measures are continued, the global AEMT-related mortality rates, DALYs, years of disability (YLDs) and YLLs are expected to increase by 2035, but it is necessary to be alert to potential new risks in the context of medical technology innovation and strengthen The medical regulatory system and risk prevention and control mechanism to achieve a reduction in the fairness of the burden (Figure 11).

## Conclusion

From 1990 to 2021, the global burden of disease attributable to adverse effects of medical treatment (AEMT) has consistently declined in terms of age-standardized mortality rates, Disability-Adjusted Life Years (DALYs), and Years of Life Lost (YLLs). However, trends in Years Lived with Disability (YLDs) indicate that while the burden of disability initially increased, it has since stabilized or slightly decreased. The reduction in AEMT-related burden has been observed across all Socio-Demographic Index (SDI) categories, with low and middle SDI countries experiencing the most significant improvements. Although high SDI countries maintain relatively low rates, they have shown a slight increase in Age-Standardized Death Rates (ASDR) and YLDs in recent years, highlighting the need for ongoing attention to patient safety and healthcare quality in these regions. The global decline in disease burden validates the efficacy of public health interventions; however, disparities in SDI reveal systemic inequities in resource allocation. Key priorities include: The global decline in disease burden emphasizes the necessity of tailored public health strategies across different SDI categories. Low-SDI countries must prioritize strengthening infectious disease control and primary healthcare systems through international collaboration to address persistent gaps in healthcare access and quality (Willis et al., 2022). Conversely, high-SDI countries should focus on innovating chronic disease management to tackle aging-related health challenges and mitigate the risks associated with surgical overuse. Meanwhile, middle-SDI countries can sustain their progress by scaling hybrid strategies, such as public-private partnerships and digital health solutions, to optimize resource allocation and healthcare delivery. These targeted approaches are essential for addressing the unique challenges faced by each SDI group and ensuring equitable health outcomes globally.

Future research must quantify socioeconomic determinants (e.g., education, health expenditure) and integrate clinical data (e.g., types of surgery, complication profiles) to refine policy targeting (Li et al., 2021; Jiang et al., 2017; Lopez et al., 2023). Mortality trends, as a nexus of technical capacity and risk trade-offs, should be embedded within multidimensional health outcome frameworks to guide equitable global health strategies. While global reductions in disease burden validate the efficacy of public health interventions, analyses stratified by Socio-Demographic Index (SDI) expose structural inequities in health outcomes (Margariti et al., 2023). To address these disparities, low-SDI countries must prioritize perioperative safety, infection control, and primary healthcare through international collaboration to reduce avoidable deaths. High-SDI countries should refine surgical indication criteria to mitigate the burdens of overmedicalization-driven disability and explore cost-effective chronic disease management strategies. Middle-SDI countries may adopt hybrid approaches (e.g., public-private partnerships, digital health) to balance accessibility and quality, thus avoiding the pitfalls of technology-dependent models (Ahuja, 2019; Uslu and Stausberg, 2021; Williams and Boren, 2008). Future research must integrate clinical epidemiological data (e.g., surgical complication profiles) with SDI stratification to identify precise drivers of inequity and inform targeted policy design.

Digital health technology has the potential to improve patients' accessibility, utilization rate and experience of healthcare. At the same time, their development and use will enhance, exacerbate and even cause adverse medical effects. These technologies have the potential to significantly improve care for individuals and populations. For example, a systematic review and meta-analysis of digital health interventions for hypertension management among populations with health disparities revealed that customized initiatives leveraging digital health offer significant advantages in managing hypertension (Katz et al., 2024). Simultaneously, digital health technologies may exacerbate or even cause health disparities among vulnerable groups in society, such as ethnic minorities, the elderly, and patients with low socioeconomic status who are at risk of low digital literacy. As the digital health wave progresses, it becomes increasingly challenging for these groups to access adequate medical resources (Lawrence, 2022a; Leape et al., 1991; Lee, 2014). Future research must prioritize people-centered design, ensuring that digital health solutions cover diverse populations and enable those in genuine need of medical resources to receive appropriate assistance (Lawrence, 2022b).

Moreover, excessive medicalization has placed additional strain on limited medical resources (Mittal et al., 2022). It is crucial to strike a balance between uncritically accepting the medicalization of human existence and blindly criticizing new cases of medicalization (Kaczmarek, 2022). Limited medical resources should be strategically allocated to populations bearing the heaviest disease burden, which can help reduce adverse medical events and, consequently, alleviate the global burden of diseases (Mor, 2022). Reasonable medicalization is essential for ensuring medical equity, particularly in low SDI countries, thereby reducing their disease burden.

The post-pandemic challenges of public health security and medical inequality present a complex and interwoven scenario. Studies indicate that systemic injustices in the allocation of medical resources not only intensify public health crises but also serve as potential catalysts for social unrest. For instance, in regions with weak medical systems (e.g., low SDI and economically disadvantaged countries), pandemic mortality rates were relatively higher, eroding public trust in governments and even triggering violent conflicts. Future research should further quantify the mitigating effects of medical equity on security issues. Integrating health equity into the framework of non-traditional security may provide a critical pathway to overcoming these challenges.
